# Loss of Protein Kinase Csnk2b/CK2β at Neuromuscular Junctions Affects Morphology and Dynamics of Aggregated Nicotinic Acetylcholine Receptors, Neuromuscular Transmission, and Synaptic Gene Expression

**DOI:** 10.3390/cells8080940

**Published:** 2019-08-20

**Authors:** Nane Eiber, Michael Rehman, Bojana Kravic, Rüdiger Rudolf, Marco Sandri, Said Hashemolhosseini

**Affiliations:** 1 Institute of Biochemistry, Medical Faculty, Friedrich-Alexander-University of Erlangen-Nürnberg, 91054 Erlangen, Germany; 2 Weill Cornell Medical College, Department of Medicine, New York, NY 10065, USA; 3 Faculty of Biology, University of Duisburg-Essen, 45141 Essen, Germany; 4 Institute of Molecular- and Cellular Biology, University of Applied Sciences Mannheim, 68163 Mannheim, Germany; 5 Department of Biomedical Science, University of Padova, 35122 Padova, Italy

**Keywords:** Csnk2b, Casein Kinase 2, acetylcholine receptor, neuromuscular junction

## Abstract

The protein kinase Csnk2/CK2 is important for cell proliferation, differentiation, and survival. Previously, we showed that CK2 binds distinctive proteins at neuromuscular junctions (NMJs) of mice and phosphorylates some of them. CK2 likely stabilizes clustered nicotinic acetylcholine receptors (AChRs). In the absence of the β-subunit of CK2 in skeletal muscle fibers, mice develop an age-dependent decrease of grip strength accompanied by NMJ fragmentation and impairments of neuromuscular transmission. However, the precise role of CK2β regarding the clustering of AChRs and downstream signaling at NMJs is unknown. Here, we compared conditional CK2β-deficient mice with controls and found in the mutants (1) a lower decrement of endplate potentials after repetitive stimulation and decrements of nerve-evoked compound muscle action potentials decayed more rapidly after synaptic transmission was partially blocked, (2) that their muscle weakness was partially rescued by administration of an acetylcholine esterase inhibitor, (3) fragmented NMJs and impaired AChR clustering was detected in muscles and cultured muscle cells, (4) enlarged myonuclei, (5) impaired synaptic gene expression, and (6) a high turnover rate of their AChR clusters in vivo. Altogether, our data demonstrate a role for CK2 at the NMJ by maintaining a high density of AChRs and ensuring physiological synaptic gene expression.

## 1. Introduction

The neuromuscular junction (NMJ) is a prototypical large synapse and the point of contact between motor nerve endings and a specialized surface area of muscle fibers [1]. Pre- and postsynaptic signaling pathways are mandatory for the formation and the maintenance of NMJs, and neuromuscular transmission is needed for muscle contraction. Motor nerve derived isoforms of a large heparan sulfate proteoglycoprotein called agrin are mandatory for NMJ formation and initiate a signaling cascade through interaction with their receptor Lrp4 on the surface of muscle fibers [2,3,4]. The receptor tyrosine kinase MuSK, part of a co-receptor with Lrp4, then becomes active and ensures both clustering of nicotinic acetylcholine receptors (AChRs) and postsynaptic gene expression by a small number of myonuclei positioned underneath the postsynaptic membrane [2,3,4,5,6]. A pretzel-like shape reflects the morphology of clustered AChRs of adult muscle fibers, and these pretzels serve as hallmarks for physiological NMJs. Many perturbations of NMJs lead to structural disorders and are often represented by a fragmentation of the NMJ [1]. Several intracellular MuSK binding proteins have been discovered in recent decades, helping us to understand the signals necessary to build and maintain NMJs; some examples are cited here [7,8,9,10,11]. Previously, the protein kinase CK2 was identified by in vitro assays interacting with Rapsyn, Rac1, MuSK, Dok-7, and 14-3-3γ and phosphorylating the latter three [8,12].

The protein kinase Csnk2/CK2 is a tetramer composed of two catalytically active α- and two β-subunits; the α- subunits might be replaced by α’- subunits [13]. It phosphorylates serine and threonine residues of target proteins and was reported as being important for cell proliferation, differentiation, and survival [13]. Although CK2 is expressed ubiquitously, accumulation in different cell compartments was reported [13]. A huge number of CK2 targets have been published and, as expected, constitutive CK2α or CK2β knockout mice are embryonically lethal [14,15]. A few years ago, CK2 was identified as interacting with MuSK and phosphorylating it [8]. Importantly, in the absence of CK2β in their muscle fibers, the mice lost grip strength in an age-dependent fashion. This muscle weakness was associated with heavily fragmented NMJs and impaired neuromuscular transmission. Later, MuSK was shown to also interact with Rapsyn, Rac1, Dok-7, and 14-3-3γ at NMJs and to phosphorylate the latter two [12]. Interestingly, phosphorylation of Rapsyn was very likely in silico but not experimentally confirmed [12]. Recently, it turned out that CK2 is also extra-synaptically important in muscle fibers, and its absence is associated with a compromised oxidative metabolism, consequently targeting mainly oxidative muscle fibers [16,17]. In fact, biopsies from human patients with mitochondrial myopathies are frequently associated with different transcript or activity profiles of CK2 [18]. Mechanistically, CK2 phosphorylates Tom22, the receptor of the mitochondrial protein import machinery, and thereby ensures muscle fiber homeostasis [16].

Here, we wanted to characterize the extent of the contribution of CK2 to the structure and the function of NMJs. We carefully investigated the muscle phenotype of conditional mutant mice, which do not express CK2β in their muscle fibers, in comparison with controls. Conditional knockout of CK2β in skeletal muscles of mice was ensured by human skeletal actin (HSA) driven Cre recombinase [8]. By blocking neuromuscular transmission in a dose-dependent manner, we unraveled the importance of CK2 activity for physiological neuromuscular transmission accompanied by significant changes of postsynaptic gene expression and a high turnover rate of AChR clusters.

## 2. Materials and Methods

### 2.1. Mice Strains

Mouse experiments were performed in accordance with animal welfare laws and approved by the responsible local committees [animal protection officer, Sachgebiet Tierschutzangelegenheiten, FAU Erlangen-Nürnberg, AZ: I/39/EE006, TS-07/11, and Italian Ministry of Health, Office 5 (authorization number 1060/2015 PR)]. Mice were housed in cages that were maintained in a room with temperature 22 ± 1 °C and relative humidity 50–60% on a 12 h light/dark cycle. Water and food were provided ad libitum. Conditional CK2ß mice were described before [8]. Mice were genotyped by PCR analysis of tail biopsy DNA [8].

### 2.2. Grid Test, Treadmill Performance

For the grip strength test, 8-month-old mice were put on the grid (made of 12 mm squares of 1 mm diameter wire), and the grid was turned upside down. The grid was kept in that position 40 cm above the ground for a period of 120 sec per repetition. There were five consecutive repetitions in total without resting time between repetitions. Neostigmine administration to mice was described before [19]. Further, muscle force was measured with all four limbs by a Grip Strength Test Meter (Bioseb, Chaville, France). For assessment of physical endurance, a test based on treadmill performance was performed [20]. Mice were initially familiarized with the exercise protocol by walking or running on a rodent treadmill on consecutive days, during which they performed 20 min of exercise with 0% grade. A day later, mice were placed into a motorized wheel and allowed to run with a starting speed of 10 m per sec. After 10 min of exercise, the speed was increased by 2 m per sec each minute until the animals were exhausted. The experiment was repeated after 2, 4, and 24 h.

### 2.3. Nerve-Muscle Preparations and Extracellular Recordings

Diaphragm-phrenic nerve preparations were maintained ex vivo in Liley’s solution gassed with 95% O_2_, 5% CO_2_ at room temperature [21]. The recording chamber had a volume of approximately 1 mL and was perfused at a rate of 1 mL/min. The nerve was drawn up into a suction electrode for stimulation with pulses of 0.1 ms duration. The preparation was placed on the stage of a Zeiss Axio Examiner Z1 microscope (Carl Zeiss MicroImaging, Göttingen, Germany) fitted with incident light fluorescence illumination with filters for 547 nm/red (Zeiss filter set 20) fluorescing fluorophore (Carl Zeiss MicroImaging). At the beginning of the experiment, the compound muscle action potential (CMAP) was recorded using a micropipette with a tip diameter of approximately 10 µm filled with bathing solution. The electrode was positioned so that the latency of the major negative peak was minimized. The electrode was then positioned 100 µm above the surface of the muscle, and CMAP was recorded.

For recordings in the presence of tubocurarine, the chamber was filled with 2 mL (300 nM, 500 nM, 800 nM, or 1000 nM) of d-tubocurarine chloride (Sigma Aldrich Chemie, München, Germany). During the curare treatment, trains of 25 repetitive nerve stimulations (5 Hz) were performed at 2 min intervals, and the ratio of CMAP amplitudes (mean (20th–25th)/2nd) was calculated [22].

### 2.4. Intracellular Recordings and Data Analysis

To block muscle action potentials so that EPPs (endplate potentials) and EPCs (endplate currents) could be recorded [23,24], µ-conotoxin GIIIB (µ-CTX, 2 µM; Peptide Institute, Osaka, Japan) was added to Lilly’s solution. Concurrently, clustered AChRs at NMJs were labeled by adding 0.5 × 10^−8^ M of rhodamine-α-bungarotoxin (Life Technologies, Darmstadt, Germany) to the same Lilly solution. In some experiments, the effect of the toxin wore off after 1–2 h, and contractions resumed in response to nerve stimulation. These preparations were then exposed a second time to the toxin. Two intracellular electrodes (resistance 10–15 MΩ) were inserted within 50 µm of the NMJs under visual inspection [24]. Current was passed through one electrode to maintain the membrane potential within 2 mV of −75 mV, while voltage transients were recorded with the other. Signals were amplified by an Axoclamp 900 A and digitized at 40 kHz by a Digidata 1440 A under the control of pCLAMP 10 (Molecular Devices, Sunny Vale, CA, USA). Voltage records were filtered at 3 kHz and current records at 1 kHz (8-pole Bessel filter). Current transients were recorded using the two-electrode voltage-clamp facility of the Axoclamp 900 A. Clamp gains were usually 300–1000, reducing the voltage transients to <3% of their unclamped amplitudes. At most NMJs, 50–100 spontaneous quantal events were recorded during a period of 1 min. Records were analyzed using pCLAMP 10. Spontaneous events were extracted using the “template search” facility and edited by eye to remove obvious artifacts. Events recorded from each NMJ were averaged, and the amplitude and frequency were determined.

### 2.5. Quantitative 3D Morphometrical Imaging

Mice were killed by CO_2_ affixation. Mouse soleus muscle was dissected and fixed in 2% paraformaldehyde (PFA) for 2 h at 4 °C. Muscle bundles containing 5–10 fibers were prepared and stained with bungarotoxin (BTX) 1:2.500 (Rh-BTX, Invitrogen, Darmstadt, Germany) for 1 h at room temperature. Stained bundles were washed three times 5 min in phosphate buffered saline (PBS) and embedded in Mowiol. Then, 3D images of NMJs were taken with a 40× oil objective (Zeiss Examiner E1, Carl Zeiss MicroImaging) at 55 ms exposure time. Images were deconvoluted and analyzed using different modules in AxioVision Software (ZEISS AxioVision Release 4.8, Carl Zeiss MicroImaging)). The following parameters were determined for each NMJ: volume, surface, grey sum, grey mean, number of fragments. For each genotype, more than 50 NMJs were analyzed.

### 2.6. Tissue Sections, Immunohistochemistry

For immunofluorescence analysis, all muscles were quick-frozen in prechilled isopentane and embedded in Tissue-Tec (Leica Instruments, Wetzlar, Germany). Muscles were cryotome-sectioned to 10 μm slices. Unfixed sections were rinsed with PBS and permeabilized for 5–10 min in PBS supplemented with 0.1% Triton X-100, then blocked in 10% fetal calf serum (FCS) and 1% bovine serum albumin (BSA) for 1–2 h. Cryotome sections were used for immunofluorescence stainings. For immunofluorescent stainings, sections were washed for 5 min in PBS, permeabilized in 0.1% Triton X-100 for 10 min, and blocked in PBS solution containing 10% FCS and 1% BSA for 1 h at 25 °C. Rhodamine- or Alexa 647 conjugated bungarotoxin 1:2.500 (Rh BTX; Thermo Fisher Scientific, Schwerte, Germany) was used for NMJ detection. Stained cryosections were analyzed and documented using a Zeiss Axio Examiner Z1 microscope (Carl Zeiss MicroImaging)) equipped with an AxioCam MRm camera (Carl Zeiss MicroImaging) and ZEISS AxioVision Release 4.8 (Carl Zeiss MicroImaging). Nuclei diameters were analyzed with the Scion Image Software (Scion Corporation, Frederick, MD, USA).

### 2.7. Tissue Culture, Culturing of Primary Muscle Cells, Transfection

Primary skeletal muscle cells were prepared from leg muscles of neonatal mice. The tissue was minced with a razor blade and dissociated with collagenase type IV (Sigma Aldrich Chemie, Taufkirchen, Germany) and Dispase II (Roche Diagnostics, Mannheim, Germany) in Mg^2+^- and Ca^2+^-free Hank´s Balanced Salt Solution (HBSS) buffer. Digestion was stopped by adding FCS. After filtering through a cell strainer (Invitrogen) and centrifugation, cells were resuspended in 80% Ham’s F10 (Ca^2+^-free medium), 20% FCS, 1% penicillin/streptomycin, and recombinant human fibroblast growth factor (5 ng/mL) (Promega Corporation, Fitchburg, MA, USA). Subsequently, cells were seeded on Matrigel-coated plates (Invitrogen, Carlsbad, CA, USA). After 24 h, the culture medium was replaced by 40% Dulbecco’s Modified Eagle Medium (DMEM), 40% Ham’s F10, 20% FCS, 1% penicillin/streptomycin, and recombinant human fibroblast growth factor (5 ng/mL). As soon as primary skeletal muscle cells reached confluence, the medium was replaced for differentiation by 98% DMEM, 2% horse serum, and 1% penicillin/streptomycin.

The production of agrin-conditioned media was described previously [25]. Agrin-conditioned medium was added at 1:8 dilution to primary myotubes. AChR aggregates were detected or quantified 16 h later, as described below. The terms active and inactive reflect agrin originating either from isoform agrinA0B0 or agrinA4B8 [26].

Quantification of different AChR endplate types was performed by analyzing >50 AChR aggregates for maximal length and surface area using ZEISS Axiovision software modules AutoMeasure Plus and Commander (Carl Zeiss MicroImaging).

### 2.8. DNA and RNA Preparation, Reverse Transcription, PCR

Total RNA was extracted from mouse diaphragm, gastrocnemius, and soleus muscle with TRIzol reagent (Life Technologies, Darmstadt, Germany) [8]. After reverse transcription, cDNAs were used with mouse-specific primers (see Table 1) for quantitative PCR reactions using the ABsolute QPCR SYBR Green Capillary Mix (Thermo Fisher Scientific, Schwerte, Germany) and the glass capillaries and Lightcycler Thermal Cycle System (Roche Diagnostics, Mannheim, Germany). Data analysis was performed as described elsewhere [8].

### 2.9. In Vivo Microscopy and Analysis of AChR Turnover and NMJ Fragmentation

In vivo microscopy of mice was performed under anesthesia using Zoletil and xylazine on a Leica SP2 confocal microscope (Leica Instruments, Wetzlar, Germany) equipped with a 63 × 1.2 numerical aperture water immersion objective, essentially as described previously [27,28]. Automated analysis of AChR turnover and NMJ fragmentation used algorithms described earlier [28].

Quantification of mean intensities of BTX-AF647 or BTX-AF555 labeled NMJs were done using ImageJ (NIH). To quantify the mean intensity, each NMJ was manually cropped out from a confocal stack. Then, using the tool “sum of slices”, a projection of the stack was created. In total, >15 NMJs were analyzed of both control and CK2β-deficient muscles.

### 2.10. Statistical Analysis

Data are presented as the mean values, and the error bars indicate ± s.e.m. The significance was calculated by unpaired two-tailed t test or as indicated by the figure legends and is provided as real p-values believed to be categorized for different significance levels, such as **** *p* < 0.0001, *** *p* < 0.001, ** *p* < 0.01, or * *p* < 0.05.

## 3. Results

### 3.1. The Ablation of CKβ in Skeletal Muscle Fibers of Mice Results in Impaired Neuromuscular Transmission and Reduced Grip Strength

**Figure 1:**
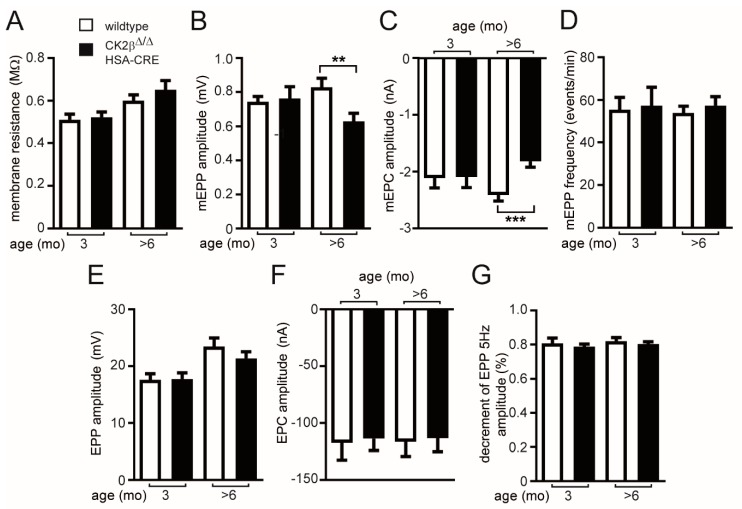
In >6-months-old mice but not 3-months-old mice, conditional CK2β-deficient mice exhibited impairment of neural transmission, as reflected by lowered miniature endplate potentials and currents in comparison with wild-type litters. The following electrophysiological recordings are presented: (**A**) membrane resistance, (**B**) miniature endplate potential amplitudes, (**C**) miniature endplate current amplitudes, (**D**) frequency of miniature endplates, (**E**) endplate potential amplitudes, (**F**) endplate current amplitudes, (**G**) decrement of endplate potential amplitude at 5 Hz. (*** *p* < 0.001; ** *p* < 0.01; unpaired two-tailed Student’s t-test; mice per genotype ≥ 3).

Previously, we recorded a difference of miniature endplate current amplitudes by comparing diaphragm muscle fibers of conditional CK2β-deficient and control mice, especially opposing control muscle fibers with regular NMJs with mutant fibers and structurally fragmented NMJs [8,16]. In order to better understand the properties of neuromuscular transmission in the CK2β-deficient mice, we measured a number of electrophysiological parameters in CK2β-deficient mutant diaphragm fibers with fragmented NMJs and control muscle fibers with unaffected NMJs. Muscle fiber action potentials were blocked by μ-conotoxin GIIIB (μCTX, 2–3 μM) to prevent contraction, as described before [29]. For these studies, we used either 3-months-old or >6-months-old mice. Next to membrane resistance, miniature endplate potentials and nerve evoked endplate potentials as well as time course and amplitudes of currents were recorded using a two-electrode voltage clamp. Although the muscles were exposed to rhodamine fluorophore coupled bungarotoxin (BTX) using a concentration of 10 nM for 30–40 min, this did not cause any significant diminution of miniature endplate potential (mEPP) amplitude (~0.8 mV, Figure 1B) compared to previously reported values [30]. We did not detect a change of membrane resistance between CK2β-deficient and control mice in either 3-months-old or in >6-months-old mice (Figure 1A). Amplitudes of nerve-independent miniature endplate potentials and currents were significantly changed in >6-months-old CK2β-deficient mice in comparison to control litters but not in 3-months-old mice (Figure 1B,C). Noteworthy, we did not detect any change in mEPP frequency in control and CK2β-deficient diaphragm muscle fibers (Figure 1D). Recording of nerve-evoked endplate potentials and currents did not reveal any changes between control and CK2β-deficient diaphragm muscle fibers (Figure 1E,F). Our first step in identifying nerve-evoked impairments failed, as we did not detect any change between control and CK2β-deficient diaphragm muscle fibers while comparing the decrement of endplate potential amplitude at 5 Hz (Figure 1G). In this case, the diaphragm muscle was stimulated by 25 sweeps within approximately 3–4 s.

**Figure 2:**
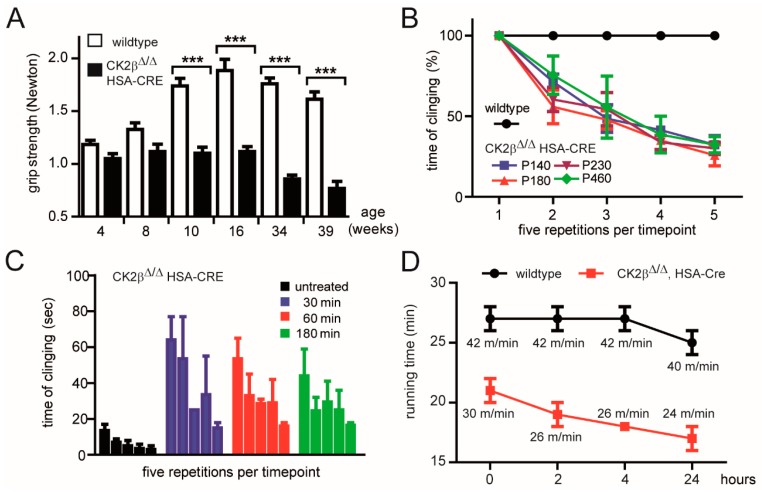
Grip strength of muscle-specific CK2β-deficient mice. (**A**) Grip strength of >5 different pairs of mice (each mouse pair was composed of wild-type and mutant litters) of indicated age was measured by a grip-strength meter (Bio-GS3 Grip-Test, Bioseb Vitrolles, France). (*** *p* < 0.001; unpaired two-tailed Student’s t-test; *n* = >5 mice, each genotype). Error bars indicate s.e.m (**B**) Muscle grip strength was determined five consecutive times by measuring the time mice were able to cling upside-down on a grid until they fell down. Mice were then immediately put back on the grid, and the drop time was measured again. The first maximal clinging time measurement was set to 100%. The next four maximal clinging times decreased continuously. Different colors symbolize different mouse ages of CK2β-deficient mice. As indicated, 140 up to 480 days old mice were analyzed. (**C**) Muscle grip strength was measured after administration of acetylcholine esterase inhibitor (neostigmine) to CK2β-deficient mice (tail vein injection). After indicated times, maximal upside-down clinging time was measured and plotted to the number of repetitions. Note that wild-type mice were able to cling up to 10 min upside-down on a grid, while mutant mice after neostigmine injection were only able to cling upside-down for about 50 s. (**D**) Endurance capacity of aged-matched wild-type and mutant mice (8–10 months of age) was determined by enforced running on a treadmill. Note that mutant mice were exhausted at lower treadmill speed.

We wanted to understand the extent of muscle weakness in response to compromised neuromuscular transmission in more detail. We conducted several behavioral studies comparing control and conditional CK2β-deficient mice. Compared with control littermates, conditional mice that lacked the β-subunit of CK2 in skeletal muscle fibers had a loss of grip strength that started by approximately three months of age and did not further progress after an additional 6–12 weeks (Figure 2A,B). Loss of grip strength was measured by either a grip-strength meter (Figure 2A) or by counting the time mice were able to cling upside down on a grid (Figure 2B,C); grip strength loss was even more prominent after repetitive upside-down clinging on a grid (Figure 2A,B). Further, the grip strength of mutant mice could only be partly rescued by intravenous administration of the acetylcholine esterase inhibitor neostigmine (Figure 2C). In accordance with these data, conditional CK2β-deficient mice fatigued earlier during running wheel endurance training in comparison with control mice (Figure 2D). Overall, these data demonstrate a compromised neuromuscular transmission but do not disclose how nerve-evoked muscle weakness occurs.

### 3.2. In the Absence of the CK2β Subunit the Function of the NMJ Is Impaired

**Figure 3:**
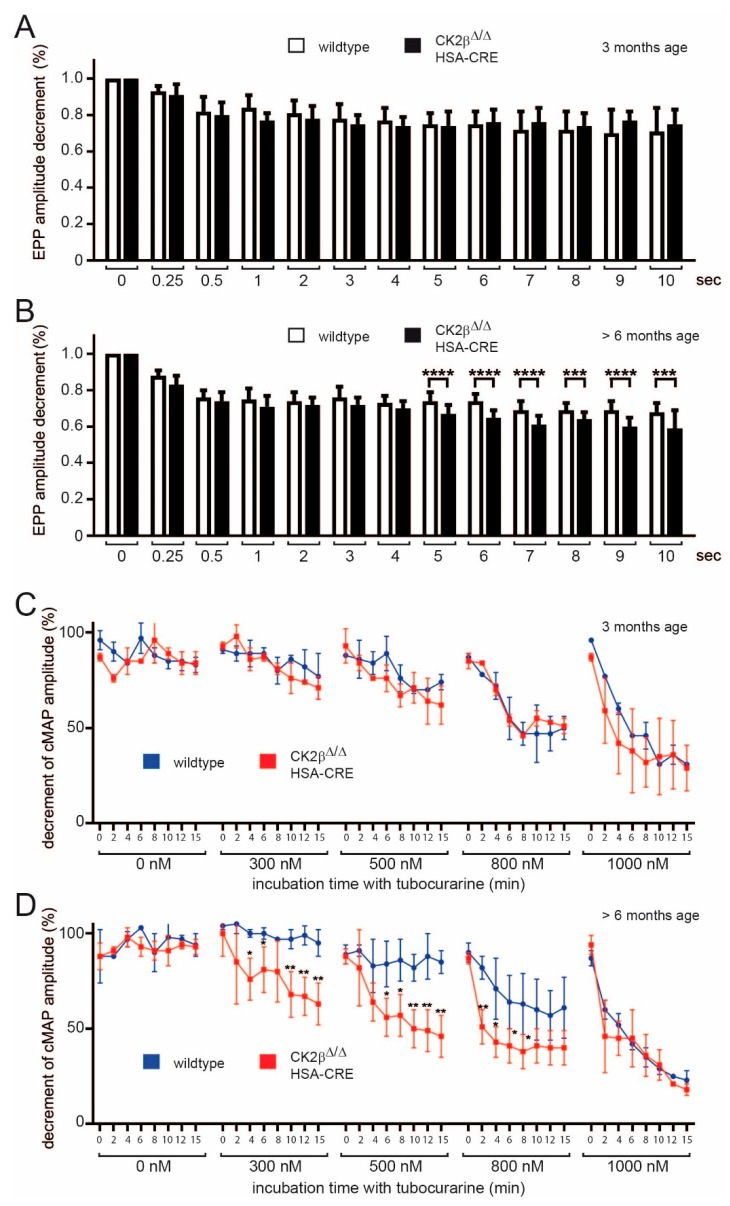
CK2β-ablation in skeletal muscles altered neuromuscular synapse function and affected safety factor in >6-months-old but not in 3-months-old conditional CK2β deficient mice. (**A**,**B**) Decrement of the endplate potential amplitude in wild-type and CK2β-deficient diaphragm muscles in 3-months-old mice (**A**) and >6-months-old mice. (**C**,**D**) Compound muscle action potential (CMAP) measurements in 3-months-old (**C**) and >6-months-old (**D**) wild-type and CK2β-deficient diaphragms under untreated conditions and in the presence of increasing concentrations (300, 500, 800, and 1,000 nM) of d-tubocurarine. The >6-months-old CK2β-deficient animals showed a significantly higher decrement of CMAP, which was already displayed at 300 nM d-tubocurarine, thus unmasking a reduced safety factor in the absence of CK2β (*** *p* < 0.001; ** *p* < 0.01; * *p* < 0.05; unpaired two-tailed Student’s t-test; *n* = 3–5 mice, each genotype). Error bars indicate s.e.m.

To better understand muscle fatigability, reduced nerve-independent changes of miniature endplate potentials, and currents in conditional CK2β-deficient mice in comparison with controls, further electrophysiological recordings were performed with phrenic nerve-diaphragm explants of wild-type and mutant mice. The reduced mEPP/miniature endplate current (mEPC) amplitudes could have been due to either decreased quantal size or to a reduced postsynaptic sensitivity to individual quanta. Notably, both above-mentioned neuromuscular transmission defects were observed, as the mean quanta were significantly reduced in CK2β-deficient muscles (data not shown). We next assessed neuromuscular transmission by repetitive stimulation of the phrenic nerve with 20 Hz trains for 10 s and calculated the decrement of EPP (Figure 3A,B). We did not measure any decrease of decrement in 3-months-old mice (Figure 3A), but there was a significant decrease of decrement starting after 5 s in >6-months-old mice (Figure 3B). We finally evaluated the safety factor, which reflected the fact that the threshold required to generate a muscle action potential was exceeded by the excitatory effect generated by nerve stimulation [31]. Towards this aim, we carried out compound muscle action potential (CMAP) measurements on wild-type and CK2β-deficient diaphragms in the presence of increasing concentrations of d-tubocurarine in order to monitor the effect of a partial block of AChRs. Treatment with d-tubocurarine led to a dose-dependent strong decrease of the decrement in response to repetitive stimuli in CK2β-deficient muscles but not in wild-type muscles in >6-months-old mice (Figure 3C,D), pointing at a reduced safety factor in CK2β-deficient mice. Obviously, a concentration range of 300–800 nM tubocurarine resulted in a stronger decrease of the decrement in mutant muscles, while high concentrations of tubocurarine (≥1000 nM) were presumably blocking too many AChRs, making it impossible to detect a change between controls and mutants (Figure 3D). Importantly, diaphragms of 3-months-old mice were similarly sensitive to tubocurarine regardless of their genotype (Figure 3C). These results indicate that lack of CK2β in muscle fibers impairs neuromuscular synaptic transmission by affecting both sustained neurotransmitter release and safety factor.

### 3.3. Structural Changes of Clustered AChRs in CK2β-Deficient Myofibers and Cultured Myotubes

**Figure 4:**
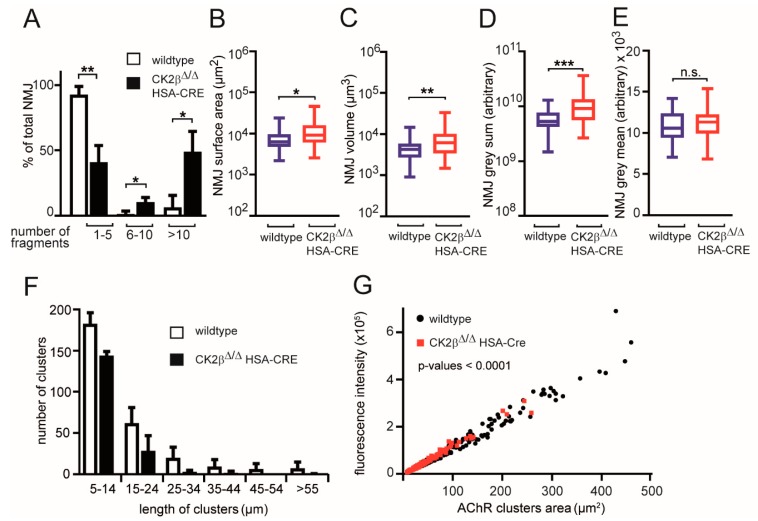
Neuromuscular junctions (NMJ) morphometric analysis of wild-type and CK2β-deficient skeletal muscles and myoblasts. (**A**) Morphometric analysis of whole-mount bungarotoxin (BTX) staining of muscles of wildtype and CK2β-deficient mice. Mutant muscles lacking CK2β displayed a significantly higher NMJ fragmentation when compared to wild-type muscles; (** *p* < 0.01; * *p* < 0.05; unpaired two-tailed Student’s t-test; *n* > 50 NMJs per genotype from at least three mice per genotype). Error bars indicate s.e.m. (**B**–**E**) Morphometric analysis of surface area (**B**), volume (**C**), total fluorescence intensity (**D**), and mean fluorescence intensity (**E**) of NMJs in whole-mount preparations of soleus of wild-type and conditional CK2β-deficient mice following staining with BTX. All the analyzed NMJ features were significantly altered in CK2β-deficient muscles except for mean fluorescence intensity (**E**) when compared to wild-type muscles (*** *p* < 0.001, ** *p* <0.01; *, *p* <0.05; n.s. means not significant; unpaired two-tailed Student’s t-test; soleus wild-type; *n* > 50 NMJs, CK2β-deficient soleus *n* > 50 NMJs). NMJs were sampled from three mice per genotype. Error bars indicate s.e.m. (**F**, **G**) Morphometric analysis of length (**F**) and fluorescence intensity per area (**G**) was performed with clustered and BTX stained acetylcholine receptors (AChRs) on cultured primary myotubes. Clusters were sampled from three primary muscle cell extracts per genotype. Error bars indicate s.e.m.

**Figure 5:**
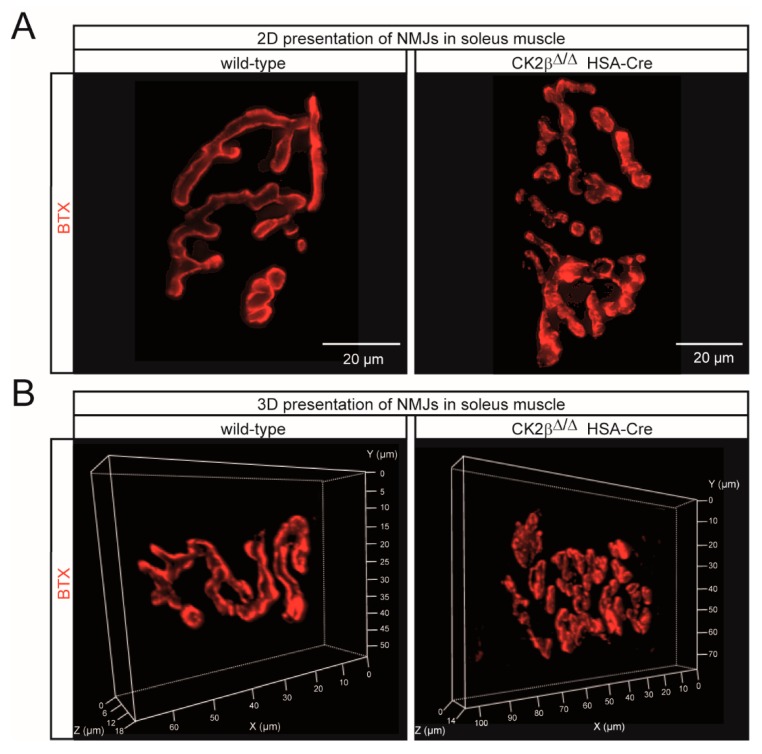
Conspicuous morphological alterations were visible at the NMJs of CK2β-deficient skeletal muscles. (**A**, **B**) Representative 2D (**A**) and 3D (**B**) images of whole-mount stainings of wild-type and CK2β-deficient soleus muscle after BTX staining (red) showing the presence of irregular and fragmented NMJs in CK2β-deficient muscles. Scale bar, 20 μm.

To explore the in vivo role of CK2β at the NMJ, we performed whole-mount BTX stainings of adult >6-months-old wild-type and mutant mouse muscles. Postsynaptic boutons of CK2β muscles appeared less regular in shape and frequently fragmented (Figure 4A and Figure 5A,B). Fragmentation was confirmed by morphometric analysis of the soleus muscle, revealing a marked fragmentation of NMJs in CK2β-deficient muscles when compared to wild-type muscles (Figure 4A). In CK2β-deficient soleus, more than half of the NMJs were assembled from more than five fragments in comparison with controls (Figure 4A). In the soleus muscle, NMJ fragmentation was accompanied by changes in 3D morphometric parameters with an increase of surface area, volume, and fluorescence intensity of NMJs (Figure 4B–E and Figure 5A,B). Endplate fragmentation was often due to aging or muscle denervation [32,33]. We asked whether these changes of AChR clustering might have been imitated in cultured cells. In accordance with our previously published data [8], treatment of primary cultured myotubes from CK2β-deficient skeletal muscles by agrin-conditioned media induced formation of aggregated AChRs of significantly smaller size in comparison with controls (Figure 4F,G).

### 3.4. Changes of Postsynaptic Gene Expression Unraveled in CK2β-Deficient Muscle Fibers

**Figure 6:**
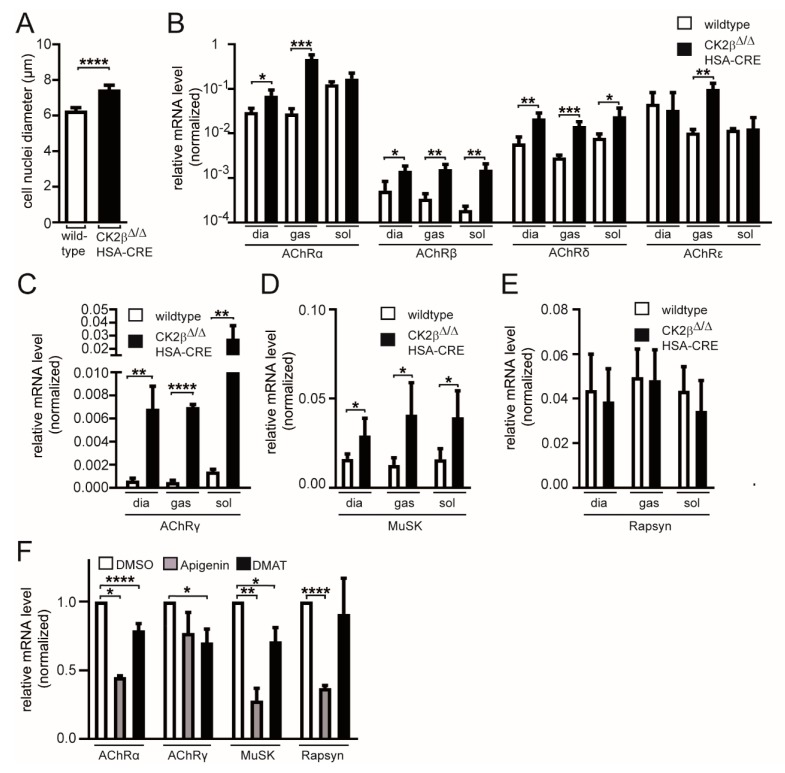
Gene expression levels for postsynaptic genes in the absence of CK2β. (**A**) Analysis of cell nuclei diameter in soleus muscle of wild-type and conditional CK2β-deficient mice. Analyzed cell nuclei were significantly bigger in diameter in CK2β-deficient muscles when compared to wild-type cell nuclei (**** *p* < 0.0001; unpaired two-tailed Student’s t-test; soleus wild-type; *n* > 50 cell nuclei, CK2β-deficient soleus *n* > 50 cell nuclei). Cell nuclei were sampled from three mice per genotype. (**B**–**E**) Relative transcript levels for AChRα, AChRβ, AChRδ, AChRε (**B**), AChRγ (**C**), MuSK (**D**), and Rapsyn (**E**) in diaphragm, gastrocnemius, and soleus muscle, as determined by quantitative PCR. Relative transcript levels of AChRβ, AChRδ (**B**), AChRγ (**C**), and MuSK (**D**) were significantly elevated in diaphragm, soleus, and extensor digitorum longus (EDL) (***p < 0.001, ** *p* < 0.01; * *p* < 0.05; unpaired two-tailed Student’s t-test; *n* > 3 muscles per genotype). (**F**) After incubation of C2C12 muscle cells with common CK2 inhibitors Apigenin and DMAT, the transcript levels of typical postsynaptic genes were significantly reduced (**** *p* < 0.0001, *** *p* < 0.001, ** *p* < 0.01; * *p* < 0.05; unpaired two-tailed Student’s t-test; *n* = 3 cell sets).

We wondered whether these structural changes of the postsynaptic machinery in CK2β-deficient fibers were accompanied with molecular differences in postsynaptic gene transcription. It is known that transcriptional activity might change the size of nuclei [34]. To gain a first insight, we analyzed muscle cell nuclei diameters. By comparing the cell nuclei diameters of CK2β-deficient and control muscle fibers, we detected a strong increase in the size of nuclei in mutant fibers (Figure 6A). Next, we looked at transcript levels of AChR subunits by comparing diaphragm, soleus, and gastrocnemius muscles of CK2β-deficient mice with controls (Figure 6B,C). Importantly, mRNA levels of AChRβ and AChRδ subunits were markedly increased in mutant muscles, while AChRα and AChRε were increased in some of the muscles (Figure 6B). We never observed a decrease in mRNA levels regarding AChR genes in CK2β-deficient muscles (Figure 6B,C). Moreover, CK2β-deficient muscles displayed a remarkable upregulation of AChRγ and MuSK, but not Rapsyn or mRNA transcripts (Figure 6C–E), thus confirming NMJ alterations and pointing to an abnormal neuromuscular transmission [35]. In order to better understand the influence of CK2 activity on the expression of postsynaptic genes, we also performed cell culture studies with two different common CK2 inhibitors, Apigenin and DMAT, which were used successfully before with cultured muscle cells [8]. It turned out that both inhibitors had a negative influence on the expression of AChRα, AChRγ, MuSK, and Rapsyn; some of these changes were evidenced as being statistically significant (Figure 6F).

### 3.5. The Turnover of Neurotransmitter Receptor Clusters Is Impressively Increased in CK2β-Deficient Muscles

**Figure 7:**
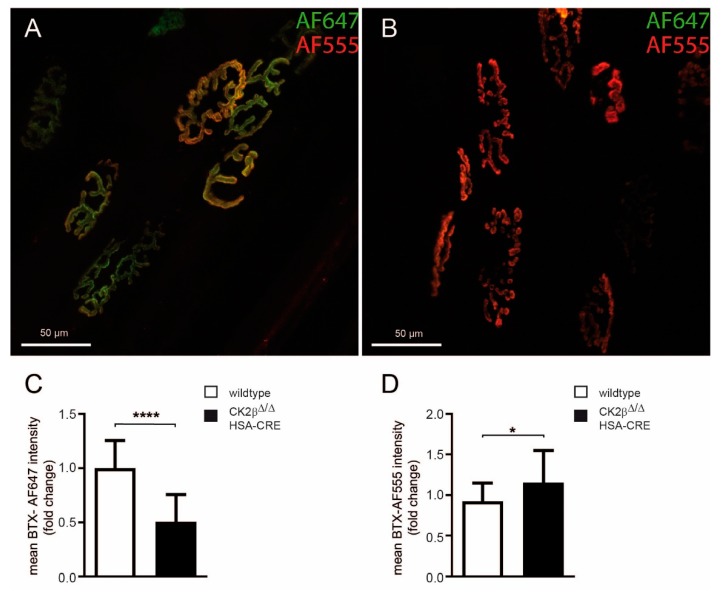
NMJs became fragmented and showed a high turnover of AChRs in CK2β-deficient muscles. (**A**,**B**) In vivo microscopy and analysis of AChR turnover and NMJ fragmentation in soleus muscle of control (**A**) and conditional CK2β-deficient (**B**) mice. TA muscles of 6–9 months old control and conditional CK2β-deficient mice were studied. Simultaneously, muscles were locally injected with BTX-AF647. Ten days later, muscles were locally injected with BTX-AF555 and then imaged using in vivo confocal microscopy. Maximum z-projections of 45 (CK2β-deficient, **B**) and 55 (control, **A**) optical slices were taken at 1.5 µm interslice distance encompassing the entire extensions of shown NMJs in depth. Overlay image, BTX-AF647 (green), BTX-AF555 (red), colocalization (yellow). Scale bar, 50 µm. (**C**,**D**) These panels show the comparison of mean intensity of either BTX-AF647 or BTX-AF555 labeled NMJs in control and wild-type and CK2β-deficient muscles. (**** *p* < 0.0001; * *p* < 0.05; unpaired two-tailed Student’s t-test; >15 NMJs were analyzed of both control and CK2β-deficient muscles.

We asked whether the absence of CK2β influenced AChR stability, as was suggested before by the disappearance of agrin-induced AChR clusters after the withdrawal of agrin but in the presence of CK2 inhibitors in cell culture [8]. Here, we decided to monitor the AChR turnover in CK2β-deficient muscles directly. Therefore, tibialis anterior (TA) muscles of control and CK2β-deficient mice were injected with BTX-AF647. Ten days later, the muscles were injected with BTX-AF555. Then, BTX-AF647 (old AChR) and BTX-AF555 (new AChR) fluorescence signals were monitored by in vivo confocal microscopy. Previously, it was reported that, given the extreme stability of the AChR-BTX complex and the small quantities of BTX (25 pmol) injected at each occasion, the largest proportion of BTX should have been available in the circulatory system only for a limited amount of time. Therefore, AChRs marked by BTX should have been surface-exposed at the time point of injection, i.e., ten days or about one hour before inspection for BTX-AF647 or BTX-AF555, respectively [27]. In control muscles, the two BTX fluorescence signals showed a very significant overlap and clearly depicted the typical arborized structure of adult NMJs (Figure 7A). In CK2β-deficient muscles, the overall architecture of NMJs was significantly altered and appeared fragmented (Figure 7B). Furthermore, although the intensity and the extension of the “new receptors” were still comparable to the control muscles (Figure 7A), “old receptors” were completely gone in CK2β-deficient muscles (Figure 7B). These changes were reflected by the quantities of “old” and “new” receptors in the muscles (Figure 7C,D). While the amount of BTX-AF647 was lowered in CK2β-deficient NMJs (Figure 7C), the amount of BTX-AF555 was significantly increased in CK2β-deficient NMJs (Figure 7D). In summary, analyzed CK2β-deficient NMJs displayed both a higher fragmentation and an increased AChR turnover rate.

## 4. Discussion

The protein kinase CK2 was shown to play a substantial role in adult muscle fibers and at NMJs by interacting and/or phosphorylating members of the protein import machinery of mitochondria or members of the postsynaptic apparatus [8,12,16,17]. Since we used a conditional CK2β-deficient mouse model and ensured proper deletion of CK2β by HSA-Cre reporter mice, it is likely that the changes we observed in the mutant mice took place in an age-dependent manner at more than 2–3 months. In fact, our studies targeted the role of CK2 in adult muscle fibers and did not analyze the influence of CK2 in early myogenesis or injury-induced muscle regeneration.

Here, we wanted to explore the structural and the functional links between CK2 and NMJs in more detail. The differences in neuromuscular transmission between CK2β-deficient muscles and controls appeared to be rather small, correlated with the same ability of mutant mice regarding voluntary walking controls in an age-independent manner [16]. Yet, we detected a significant loss of grip-strength in conditional CK2β-deficient mice in comparison with controls (Figure 2A,B), as well as a higher degree of fatigue (Figure 2B,D). These behavioral changes in mutant mice were accompanied by structural and functional changes. First, we detected a higher fragmentation grade of NMJs in mutants in comparison with controls (Figure 4 and Figure 5). Second, we recorded lower amplitudes of miniature endplate potentials and currents (Figure 1B,C), and higher fatigability, as evidenced by lower decrements of endplate potential amplitudes after run-down experiments (Figure 3B). Neuromuscular transmission impairments might have been masked by the safety factor [31]. Remarkably, we used our established ex vivo electrophysiological recording setup for measurements in the presence of increasing tubocurarine concentration to detect a prominent and significant decrease of the decrements of the compound muscle action potential in mutant diaphragms in comparison with controls (Figure 3D) [36]. Third, further information was also provided on the distribution and the size of differentially labeled pools of AChRs (Figure 4 and Figure 7). BTX stainings of newly arriving or old receptors showed their highest densities at the rim or the center of NMJs, respectively [37]. For NMJs of CK2β-deficient muscles, fluorescence signals of old receptors disappeared, arguing for a very high turnover of AChR clusters in mutant mice.

We wondered what the reasons for the rise of the AChRs turnover were. Presumably, the proven phosphorylation of the postsynaptic proteins MuSK, Dok-7, and 14-3-3γ was functionally significant at NMJs [8,12]. However, what about the expression profiles of postsynaptic genes? They might also have been compromised, either due to direct influences on their expression or because of regulatory compensation reactions. The significant increase of myonuclei diameter in muscle fibers of adult CK2β-deficient mice already pointed to an eventual increase of transcriptional activity (Figure 6A). Indeed, we detected a strong increase of transcriptional activities of AChR subunits α, β, δ, and ε (Figure 6B) in addition to AChRγ, although this increase might have been more related to regeneration events rather than a direct consequence of CK2β ablation (Figure 6C). This increase in the transcriptional rate of AChRs was in agreement with the amount of AChR protein (Figure 7D). The quantity of the new receptor (BTX-AF555) was significantly increased in NMJs of CK2β-deficient muscles (Figure 7D). Interestingly, transcript levels of MuSK were also increased, while Rapsyn transcription was not affected (Figure 6D,E). These findings might explain why MuSK was detected being phosphorylated by CK2 but not Rapsyn [8,12]. On the other hand, the transcript levels of Rapsyn also appeared to be a little lower in CK2β-deficient muscles in comparison with controls, although these decreases were not statistically significant (Figure 6E). Unexpectedly, transcript levels of postsynaptic genes were lower in cultured myotubes, which were incubated with two different CK2 inhibitors, apigenin and DMAT (Figure 6F). This discrepancy between the expression levels of postsynaptic genes in CK2β-deficient muscles and cultured muscle cells in the presence of CK2 inhibitors might be explainable, keeping in mind the different turnover times of clustered AChRs of 4–8 h in vitro in cultured myotubes in comparison to two weeks in vivo.

In this study, we did not want to analyze aging or functional denervation defects in the absence of CK2β in skeletal muscles. However, our data thus far showed no apparent change in the presynaptic compartment in CK2β-deficient muscles, which speaks against denervation defects. Nevertheless, functional denervation would be conceivable.

Overall, our data indicate that CK2 activity on NMJs is required for physiological AChR expression and AChR cluster turnover.

## Figures and Tables

**Table 1:** Oligonucleotides.

Gene	Primer Sequence
AChRα	5′-CTACCTGCCCACAGACTCAG-3′ 5′-TTGGACTCCTGGTCTGACTT-3′
AChRγ	5′-GGTCAATGTCAGCCTGAAGC-3′ 5′-GCACATGCATCCGTAACAGC-3′
AChRβ	5′-TCTCCAACTATGATAGCTCGGT-3′ 5′-CATTGATGTCCAGGGCAACGTC-3′
AChRε	5′-TGTATGGCTGCCAGAGATTG-3′ 5′-GCGGATGATGAGCGTATAGA-3′
AChRδ	5′-ATGAGGAACAAAGGCTGATCCA-3′ 5′-ACAGTGATGTTCCCGAAGTCGT-3′
Rapsyn	5′-CCGCTACAGGCACTCTGTCT-3′ 5′-TCAGTCTCCTCCACGCACTC-3′
MuSK	5′-GCCTTGGTTGAAGAAGTAGC -3′ 5′-CTTGATCCAGGACACAGATG -3′
RPL8	5′-GTTCGTGTACTGCGGCAAGA-3′ 5′-ACAGGATTCATGGCCACACC-3′
